# Back-to-back comparison of penKID with NephroCheck® to predict acute kidney injury at admission in intensive care unit: a brief report

**DOI:** 10.1186/s13054-018-1945-9

**Published:** 2018-01-29

**Authors:** Etienne Gayat, Cyril Touchard, Alexa Hollinger, Antoine Vieillard-Baron, Alexandre Mebazaa, Matthieu Legrand, on behalf of the FROG ICU study investigators, N. Deye, C. Fauvaux, A. Mebazaa, C. Damoisel, D. Payen, E. Gayat, M. Legrand, Elie Azoulay, A. S. Moreau, L. Jacob, O. Marie, M. Wolf, R. Sonneville, R. Bronchard, I. Rennuit, C. Paugam, J. P Mira, A. Cariou, A. Tesnieres, N. Dufour, N. Anguel, L. Guerin, J. Duranteau, C. Ract, M. Leone, B. Pastene, T. Sharshar, A. Fayssoyl, J.-L. Baudel, B. Guidet, Q. Lu, W. Jie Gu, N. Brechot, A. Combes, S. Jaber, A. Pradel, Y. Coisel, M. Conseil, A. Veillard Baron, L. Bodson, J.Y. Lefrant, L. Elotmani, A. Ayral, S. Lloret, S. Pily-Flouri, J. B. Pretalli, P. F. Laterre, V. Montiel, M. F. Dujardin, C. Berghe

**Affiliations:** 1 Department of Anesthesiology, Critical Care and Burn Unit, Hôpitaux Universitaires Saint Louis—Lariboisière, Assistance Publique—Hôpitaux de Paris, Paris, France; 2 Université Paris Diderot—Paris 7, Sorbonne Paris Cité, Paris, France; 3 UMR-S 942, INSERM, Paris, France; 4 University Hospital Ambroise Paré, Intensive Care Unit, Assistance Publique—Hopitaux de Paris, 26930 Boulogne-Billancourt, France; 5 Department of Anesthesiology and Intensive Care, INSERM UMR-S 942, Saint Louis—Lariboisière University Hospitals, 2 rue Ambroise Paré, Paris, 75010 France

Acute kidney injury (AKI) is a frequent condition in critically ill patients that affects both short- and long-term outcome [1]. Its early detection remains a challenge, and diagnosis frequently occurs too late with cell damage already present. Implementation of novel biomarkers that reliably identify patients at risk or at an early stage of AKI could offer more efficient management strategies leading to better outcomes.

In our investigation, we compared two promising AKI biomarkers: a marker of tubular injury commercialized as a lateral-flow test (NephroCheck®) [2], the product of urinary TIMP-2 (Tissue inhibitor of metalloprotease), and IGFBP 7 (Insulin-like growth factor binding protein; overall TIMP2xIGFBP7), and the filtration marker proenkephalin A 119-159 (penKid). penKid has been recently described as a valuable plasma biomarker of AKI in the acutely ill, including septic patients [3] and patients suffering from acute heart failure [4]. Proenkephalin represents a stable surrogate analyte of labile enkephalins, which are known as endogenous opioids, but also affects kidney function [5]. The aim of our study was to conduct a parallel assessment of the two biomarkers in an intensive care unit (ICU) population from the data of the FROG-ICU study.

FROG-ICU has been designed to better understand long-term outcome after ICU discharge as well as risk factors for all-cause and cardiovascular morbidity and associated mortality (FROG-ICU study, ClinicalTrials.gov identifier NCT01367093). It was a large prospective multicenter cohort study with biological (plasma and urine) collection and one-year follow-up of ICU patients. FROG-ICU aims to allow risk stratification of ICU survivors in order to recognize the subset of patients who may benefit from early intervention to allow decreased cardiovascular morbidity and related mortality. The methodology of the FROG-ICU study was published in 2015. From the FROG study including 2087 critically ill patients we randomly selected 200 requiring mechanical ventilation or vasopressor support for more than 24 h with respect to the four categories of the renal Sequential Organ Failure Assessment (SOFA) score (i.e., creatinine level on admission of < 1.2 mg/dL (*n* = 80), 1.2 to 1.9 mg/dL (*n* = 40), 2 to 3.4 mg/dL (*n* = 40), and ≥ 3.5 mg/dL (*n* = 40)); 78% were male, with a median age of 65 years (interquartile range (IQR) 54–75). In the investigated cohort of a standard population of critically ill, the main cause of ICU admission was septic shock (26%), the median age was 65 years (IQR 54–75), the median SAPS II score was 51 (IQR 41–68), and the ICU mortality was 26%. Other causes of admission include out-of-hospital cardiac arrest (10%), acute respiratory failure (16%), acute neurological disorder (11%), and cardiogenic shock (8%). On ICU admission, the median (and IQR) value of estimated glomerular filtration rate for penKid and TIMP2xIGFBP7 was 47 mL/min (22–88), 85 pmol/L (48–40), and 0.6 UNIT (0.3–2), respectively.

**Fig. 1:**
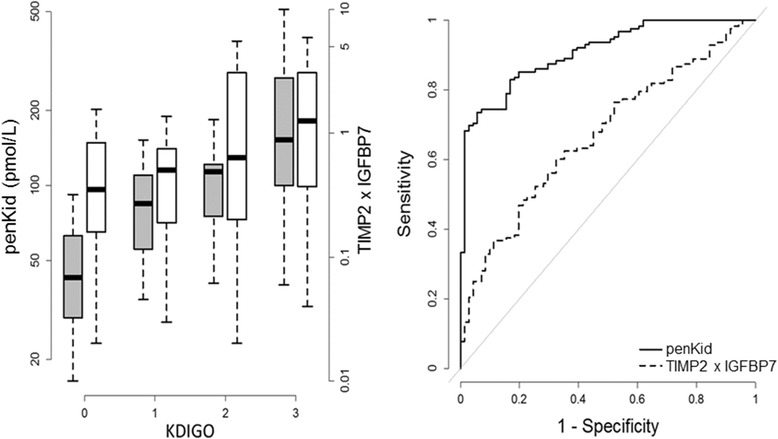
Boxplot of penKid (*grey*) and NephroCheck (*white*) levels according to severity of AKI as defined by KDIGO (*left panel*) and respective ROC curves predicting AKI (*right panel*)

AKI was defined using the Kidney Disease Improvement Global Outcome (KDIGO) definition. Accordingly, we used both the variation in serum creatinine during the first 48 h after ICU admission and the maximal value during the 7 days following ICU admission. Admission serum creatinine was used as baseline serum creatinine when the estimated glomerular filtration rate (eGFR) was above 60 ml/min/1.73 m^2^ at admission. Otherwise (*n* = 117, 59% of the study population), baseline serum creatinine was extrapolated considering a baseline eGFR of 75 ml/min/1.73 m^2^ (based on the MDRD (Modification of Diet in Renal Disease) equation). Both penKid and TIMP2xIGFBP7 were measured on ICU admission in plasma and urine. We assessed the conformity of penKid and TIMP2xIGFBP7 for prediction or detection of AKI as defined by the KDIGO classification, with the renal SOFA score, by the area under the ROC curve. On ICU admission, the median (and IQR) value of eGFR for penKid and NephroCheck® was 47 mL/min/1.73 m^2^ (22–88), 85 pmol/L (48–40), and 0.6 UNIT (0.3–2), respectively. Figure 1 shows correlation of penKid and TIMP2xIGFBP7 levels on ICU admission with the severity of AKI, and confirms that penKid as a filtration marker shows a significantly higher association with AKI (ROC curve 0.668 (95% CI 0.589–0.743) vs 0.908 (95% CI 0.868–0.944), *p* < 0.0001). When investigating renal replacement therapy (RRT) as an outcome parameter, elevated penKid levels were able to more accurately predict need of RRT in our standard ICU population (*n* = 60) when compared to elevated TIMP2xIGFBP7 levels (AUC [95% CI] 0.778 [0.713–0838] and 0.678 [0.597–0.761]). Limitations of our investigation include a possible sampling bias within the investigated population as we didn’t measure the two biomarkers in the whole cohort of 2087 patients.
